# Influenza vaccines for preventing cardiovascular disease

**DOI:** 10.1590/1516-3180.20151334T2

**Published:** 2015-08-03

**Authors:** Christine Clar, Zainab Oseni, Nadine Flowers, Maryam Keshtkar-Jahromi, Karen Rees

## Abstract

**BACKGROUND:**

This is an update of the original review published in 2008. The risk of adverse cardiovascular outcomes is increased with influenza-like infection, and vaccination against influenza may improve cardiovascular outcomes.

**OBJECTIVES:**

To assess the potential benefits of influenza vaccination for primary and secondary prevention of cardiovascular disease.

**METHODS:**

*Search methods:* We searched the following electronic databases on 18 October 2013: The Cochrane Library (including Cochrane Central Register of Controlled Trials (CENTRAL), Database of Abstracts of Reviews of Effects (DARE), Economic Evaluation Database (EED) and Health Technology Assessment database (HTA)), MEDLINE, EMBASE, Science Citation Index Expanded, Conference Proceedings Citation Index - Science and ongoing trials registers (www.controlled-trials.com/ and www.clinicaltrials.gov). We examined reference lists of relevant primary studies and systematic reviews. We performed a limited PubMed search on 20 February 2015, just before publication.

*Selection criteria:* Randomised controlled trials (RCTs) of influenza vaccination compared with placebo or no treatment in participants with or without cardiovascular disease, assessing cardiovascular death or non-fatal cardiovascular events.

*Data collection and analysis:* We used standard methodological procedures as expected by The Cochrane Collaboration. We carried out meta-analyses only for cardiovascular death, as other outcomes were reported too infrequently. We expressed effect sizes as risk ratios (RRs), and we used random-effects models.

**MAIN RESULTS:**

We included eight trials of influenza vaccination compared with placebo or no vaccination, with 12,029 participants receiving at least one vaccination or control treatment. We included six new studies (n = 11,251), in addition to the two included in the previous version of the review. Four of these trials (n = 10,347) focused on prevention of influenza in the general or elderly population and reported cardiovascular outcomes among their safety analyses; four trials (n = 1682) focused on prevention of cardiovascular events in patients with established coronary heart disease. These populations were analysed separately. Follow-up continued between 42 days and one year. Five RCTs showed deficits in at least three of the risk of bias criteria assessed. When reported (seven studies), vaccination provided adequate immunogenicity or protection against influenza. Cardiovascular mortality was reported by four secondary prevention trials and was significantly reduced by influenza vaccination overall (risk ratio (RR) 0.45, 95% confidence interval (CI) 0.26 to 0.76; P value 0.003) with no significant heterogeneity between studies, and by three trials reporting cardiovascular mortality as part of their safety analyses when the numbers of events were too small to permit conclusions. In studies of patients with coronary heart disease, composite outcomes of cardiovascular events tended to be decreased with influenza vaccination compared with placebo. Generally no significant difference was found between comparison groups regarding individual outcomes such as myocardial infarction.

**AUTHORS’ CONCLUSIONS:**

In patients with cardiovascular disease, influenza vaccination may reduce cardiovascular mortality and combined cardiovascular events. However, studies had some risk of bias, and results were not always consistent, so additional higher-quality evidence is necessary to confirm these findings. Not enough evidence was available to establish whether influenza vaccination has a role to play in the primary prevention of cardiovascular disease.

**This abstract is available free of charge from:** http://onlinelibrary.wiley.com/doi/10.1002/14651858.CD005050.pub3/abstract.
